# Dr. Sutton on Typhoid Fever

**Published:** 1850-09

**Authors:** 


### DR. SUTTON ON TYPHOID FEVER.

We commence in this number, the publication of an admirable monograph on typhoid fever, from the pen of Dr. Sutton, of Georgetown, Kentucky. It is scarcely necessary for us to commend strongly to the attention of the reader, an essay that will at once command his respect, as soon as he commences its perusal. It is written in that philosophical spirit that should characterise all medical writing, and is full of devotion to truth, of correct and careful observation, and fair and legiitmate inferences. The disease, to which Dr. Sutton's monograph is devoted, has attracted much attention from medical men, and those who are familiar with the malady, will find no difficulty in realising that Dr. Sutton handles it with the power of a full acquaintance with the subject. Those who are not yet familiar with typhoid fever, may make themselves so by a diligent study of this essay. We shall probably complete it in the November number of this Journal. B.

